# Uncomplicated oocyte donation pregnancies display an elevated CD163‐positive type 2 macrophage load in the decidua, which is associated with fetal‐maternal HLA mismatches

**DOI:** 10.1111/aji.13511

**Published:** 2021-12-04

**Authors:** Xuezi Tian, Kaveri T. S. Aiyer, Johanna M. Kapsenberg, Dave L. Roelen, Marie‐Louise van der Hoorn, Michael Eikmans

**Affiliations:** ^1^ Department of Obstetrics and Gynecology Leiden University Medical Center Leiden Netherlands; ^2^ Department of Immunology Leiden University Medical Center Leiden Netherlands

**Keywords:** decidua, HLA antigens, macrophages, oocyte donation, pregnancy

## Abstract

**Problem:**

The embryo of an oocyte donation (OD) pregnancy is completely allogeneic to the mother, which may challenge the maternal immune system to tolerize the fetus. Decidual macrophages are essential in maintaining a healthy pregnancy, and type 2 macrophages may exhibit immune suppressive activity. We hypothesized that the composition of decidual macrophages is different between uncomplicated OD pregnancies and non‐OD in vitro fertilization (IVF) pregnancies, and is related to fetal‐maternal incompatibility.

**Method of study:**

Women with uncomplicated pregnancy were enrolled: 25 singleton OD pregnancies and 17 non‐OD IVF pregnancies. The extent of immunohistochemical staining of CD14 (pan‐macrophage marker) and CD163 (type 2 macrophage marker) in both decidua basalis and parietalis was quantitated by digital image analysis. Maternal and fetal DNA was typed for human leukocyte antigen (HLA)‐A, ‐B, C, ‐DRB1, and ‐DQB1, and fetal‐maternal HLA mismatches were calculated.

**Results:**

OD pregnancies showed a higher percentage of CD163+ staining (*P* = .040) and higher CD163/CD14 ratio (*P* = .032) in the parietalis than non‐OD IVF. The OD group was separated into a semi‐allogeneic group (≤5 fetal maternal HLA mismatches) and a fully allogeneic group (> 5 mismatches). The HLA‐fully‐allogeneic OD group, but not the HLA‐semi‐allogeneic OD group, showed significantly elevated CD163/CD14 ratio in the parietalis compared with the non‐OD IVF group (*P* < .05).

**Conclusions:**

Uncomplicated OD pregnancies display a higher CD163‐positive cell fraction in the total decidual macrophage population compared to autologous pregnancies, which may suggest that a local type 2 macrophage‐related mechanism is needed to compensate for the higher fetal‐maternal HLA mismatch load.

## 1 INTRODUCTION

During pregnancy, fetal and maternal tissue come into contact as soon as the blastocyst implants into the endometrium and thereafter through the whole gestation period.^1^ Since the fetus is genetically different from the mother, the fetal cells are exposed to a potential attack of the maternal immune system.^2^ To maintain an uncomplicated pregnancy until term, immune modulatory mechanism are at play at the fetal‐maternal interface.^3^ Maternal immune cells contact with fetal cells at multiple locations during gestation,^4^ in which the decidua basalis is the maternal part of the placenta and invaded by the extravillous trophoblast; the decidua parietalis is the maternal part of the membrane and confronts the trophoblast cells of the chorion.

In oocyte donation (OD) pregnancy, the fetus has inherited genes from both the father and the donor: as a consequence the fetus usually is fully allogeneic to the pregnant mother.^5^ OD pregnancies are related to a higher extent of human leukocyte antigen (HLA) mismatches between the fetus and the mother compared to non‐OD pregnancies. It is reported that women with OD pregnancy have higher risk of pregnancy complications than women with non‐OD pregnancies, which include hypertensive disorders, preeclampsia, preterm birth, and low birth weight.^6^, ^7^ In successful uncomplicated OD pregnancies, HLA matching between fetus and mother was observed to be significantly higher than expected by chance.^8^ These observations suggest that a larger number of HLA mismatches is an essential factor that accounts for women with pregnancy after OD to have a higher chance of developing complications. It is hypothesized that due to this higher gene dissimilarity, the demand for immune cells in the fetal‐maternal interface of OD pregnancies is higher, in order to maintain tolerance during pregnancy.^9^

Among all immune cells in the placental bed, macrophages have shown their importance from multiple aspects. In the total decidual immune cell population, 20–25% is represented by macrophages.^10^ This quantity stays constant over the whole period of pregnancy,^11^ suggesting that macrophages have a role in each trimester. Moreover, macrophages have been suggested to be functional in multiple maternal adaptation processes.^12^ During implantation, a large amount of macrophages migrate towards the invading trophoblasts and spiral arteries, implying a role for decidual macrophages in trophoblast invasion, vascular remodeling, and placentation.^13^, ^14^ Macrophages also act as phagocytes to engulf apoptotic cells, thereby not only preventing proinflammatory reactions but also regulating the extent of fetal cells invading into the uterine wall.^15^, ^16^

The capability of macrophages to provide multiple functions during pregnancy is related to their phenotypical plasticity. Macrophages are often classified into two categories based on phenotype and characteristics, namely classically activated macrophages (M1) and alternatively activated macrophages (M2).^17^ M1 macrophages are pro‐inflammatory and regarded to be defensive against infections and tumors, whereas M2 macrophages are anti‐inflammatory exhibiting immune suppressive activity.^18^, ^19^ Cluster of differentiation (CD) 14 is a glycosylphosphatidylinositol anchored membrane protein, expressed on monocytes and macrophages, while CD163 is a glycoprotein antigen expressed on M2 macrophages.^10^, ^20^

As macrophages are essential in maintaining healthy pregnancy we hypothesize that the quantity and composition of decidual macrophages are different between uncomplicated OD pregnancies and non‐OD in vitro fertilization (IVF) pregnancies, and that these differences in macrophages are related to fetal‐maternal incompatibility. Therefore, in this study we investigated the quantity and composition of the macrophage population in those groups by immunohistochemical staining and related the macrophage load to fetal‐maternal HLA mismatches.

## 2 METHODS

### 2.1 Patient selection

This retrospective case‐control study was performed at the Leiden University Medical Center (LUMC). Patients who delivered in the hospital between January 1, 2006 and July 1, 2016 were eligible for inclusion. A total of 42 pregnancies were enrolled in this study, conceived by OD pregnancies (*n* = 25) or non‐OD IVF pregnancies (*n* = 17). Exclusion criteria were: multiplets, maternal autoimmune disease, the presence of chromosomal abnormalities, the use of immunosuppressive medication, and complications such as preeclampsia and/or preterm birth. Medical records were reviewed and clinical data were collected. Placental tissue samples were collected for staining. Indication for OD was unknown to the investigators. The study was approved by the ethics committee of the LUMC (P16.048, P08.229/228, P10.009), and informed consent of every patient was obtained.

### 2.2 Immunohistochemistry

Placental tissues were embedded in paraffin. From each placenta two paraffin blocks were selected, one from the decidua basalis and one from the decidua parietalis. Most tissue blocks were selected from the lateral part of the placenta; central blocks were used when the lateral blocks were of low quality. Sequential serial sections (4 μm) were cut, transferred to Superfrost slides (Thermo Scientific), and dried overnight at 37°C. Tissue sections were deparaffinized for 3x5 min in consecutive xylol baths, followed with rehydration in decreasing ethanol to demi‐water. After using .4% hydrogen peroxide (H_2_O_2_) to block endogenous peroxidase for 20 min, the sections were washed in demi‐water for 5 min. Then sections were pre‐treated with heated ethylenediaminetetraacetic acid‐tromethamine (Tris/EDTA) for 10 min and cooled down for 20 min, followed by 5 min washing with demi and 5 min washing with phosphate‐buffered saline (PBS). Sections were then stained with the primary antibody diluted in 1% PBS/bovine serum albumin (BSA) and incubated overnight in the dark. The two primary antibodies used were CD163 (IgG1, dilution 1:20, Abcam, Cambridge, UK) and CD14 (IgG2a, dilution 1:100, Novocasta Laboratories Ltd, Newcastle, UK), respectively. After 3x5 min washing with PBS, the slides were incubated for 30 min with the secondary antibody, Envision horseradish peroxidase (HRP) (DAKO, North America Inc, USA). Following another 3x5 min washing in PBS, the sections were incubated with diaminobenzidine (DAB, DAKO Cytomation) for 5 min. The reaction was terminated by removing the substrate and washing the sections with demi‐water. Then the sections were counterstained with hematoxylin and mounted in mounting medium (Surgipath Medical Ind, Inc. Richmond) and covered.

### 2.3 Double label immunofluorescence of CD14 and CD163

Three basalis samples and three parietalis samples with various CD163/CD14 ratios were randomly selected to perform double label immunofluorescence of CD14 and CD163. Paraffin‐embedded sections (4 μm) were deparaffinized for 3x5 min in xylol and hydrated via graded ethanol to demi‐water. Then sections were pre‐treated with heated citrate for 10 min and cooled down for 30 min, followed by washing in PBS for 5 min. Sections were incubated overnight with a mix of primary antibodies CD14 (rabbit anti‐human IgG, dilution 1:500, D7A2T, CellSignaling, USA) and CD163 (mouse anti‐human IgG1, dilution 1:20, 10D6, Abcam, UK). Thereafter, the sections were washed with PBS for 3x5 min, and incubated with a mix of secondary antibodies: Alexa Fluor 546 goat‐anti‐rabbit IgG (dilution 1:200, Life Technologies, the Netherlands) and Alexa Fluor 488 goat‐anti‐mouse IgG1 (dilution 1:200, Life Technologies). Then the sections were mounted with ProLong Gold antifade mount with 4′,6‐diamidino‐2‐phenylindole (DAPI) (70 μl/slide, Life Technologies), and stored in the dark at room temperature for approximate 2 h.

### 2.4 Quantification of immunohistochemistry staining

All slides were scanned by a Panoramic Midi scanner (serial no. PMIDI‐000271, model 3Dhistech). The scoring was performed at a 10X zoom on a 24‐inch screen. The entire decidua parietalis was scored semi‐quantitatively on a four‐point scale (0 = no staining, 1 = minimal, 2 = moderate, 3 = extensive). Since there were several basalis portions on one basalis slide and each basalis portion was scored individually using this four‐point scale, the average of all basalis portions scores was used to sort one basalis slide into three categories (mild: < 1.5, moderate: 1.5≤ ‐ < 2.5, extensive: ≥2.5). Representative pictures of different semi‐quantitatively scored slides of the decidua basalis and parietalis are shown in Figure S1A‐D and Figure S2A‐D.

The entire decidua basalis and parietalis was quantitatively analyzed using the pattern quant modus in QuantCenter software 2.2. (3Dhistech, Budapest, Hungary). The program is capable of selecting positive cells by pattern recognition and training (Figure S1E‐F, Figure S2E‐F). The extent of staining was assessed according to the percentage of positive staining in the total tissue area, which contained all the cells and tissues of decidua basalis or parietalis but not the blank area on the slides.

All basalis slides were semi‐quantitatively and quantitatively scored by KA. All parietalis slides were semi‐quantitatively and quantitatively scored by XT. Fifteen slides for decidua basalis and twenty slides for decidua parietalis were randomly selected and assessed by two investigators individually, to determine the discrepancy between two observers for both semi‐quantitative and quantitative scores. All the investigators were blinded to the clinical history of the patients.

### 2.5 Quantification of double label immunofluorescence staining

On each slide, five .5mm^2^ regions containing basalis or parietalis were selected randomly to perform cell quantification analysis, using cell segmentation approaches based on the identification of nuclei. First, probability masks were trained in Ilastik^21^ to recognize which pixels of the image belonged to the nuclei or membranes with positive staining. The nuclei probability masks were put into CellProfiler^22^ and cells final masks were created by expanding a specified distance from the nuclei. These cells masks were then combined in ImaCytE^23^ with the positive staining membranes masks, which acted as threshold. Finally, tSNE analyses were run in Cytosplore^24^ and different cells were manually clustered.

### 2.6 HLA typing

HLA typing of the mothers and children was performed by the HLA typing laboratory of the LUMC. Maternal and fetal deoxyribonucleic acid (DNA) was typed for HLA‐A, ‐B, C, ‐DRB1, and ‐DQB1 using sequence specific polymerase chain reaction (PCR) priming. The quantities of HLA mismatches were calculated manually using Microsoft Excel 2016. All HLA antigens were evaluated at split level. The HLA class I mismatches were defined as the summary of HLA‐A, ‐B, ‐C mismatches; the HLA class II mismatches were defined as the summary of HLA‐DRB1 and ‐DQB1 mismatches; the total HLA mismatches were defined as the summary of all these five HLA loci mismatches.

To determine the association between fetal‐maternal HLA mismatches and the percentage of CD14 and CD163 positive cells, OD pregnancies were divided into two groups according to the number of HLA mismatches. In the semi‐allogeneic group, the number of mismatches was not higher than half of the antigens per HLA locus, which is similar to the maximum fetal‐maternal HLA mismatches in the non‐OD IVF pregnancies. In the fully allogeneic group, the number of mismatches was higher than half of the antigens per HLA locus, which is a situation unique for OD pregnancies.

### 2.7 Data analysis

Statistical analyses were performed using SPSS statistics 25 (IBM SPSS Software, Chicago USA) and GraphPad Prism version 8 for Windows (GraphPad Software, San Diego, CA, USA). All data were non‐normally distributed. Therefore, Mann Whitney U tests and Spearman's correlation were used to analyze continuous data and the Fisher's exact test was used for nominal data. A value of *P* < .05 was considered to represent significance.

## 3 RESULTS

### 3.1 Patient characteristics

Clinical characteristics are summarized in Table 1. There were no significant differences in any of the maternal or fetal parameters between the OD group and non‐OD IVF group.

**TABLE 1:** Patient characteristics

	OD group (*N* = 25)	Non‐OD IVF group (*N* = 17)	p value
Maternal age (years)	37.0 (27.0, 49.0)	36.0 (29.0, 41.0)	ns
BMI (kg/m^2^)	21.3 (18.6, 24.8)	22.7 (18.7, 36.7)	ns
Gravidity (number)	2 (1, 7)	2 (1, 5)	ns
Parity (number)	0 (0, 2)	0 (0, 2)	ns
Spontaneous abortions (number)	0 (0, 3)	0 (0, 4)	ns
Gestation age (days)	280.5 (270.0, 296.0)	280.0 (259.0, 294.0)	ns
Highest diastole (mmHg)	80 (60, 90)	80 (70, 95)	ns
Proteinuria	No (No, +)	No (No, No)	ns
Placental weight (gram)	645.0 (360.0, 880.0)	580.0 (410.0, 800.0)	ns
Fetal male gender (number(percentage))	12 (48.0%)	7 (41.2%)	ns
Fetal birth weight (gram)	3680.0 (3080.0, 4100.00)	3450.00 (2840.0, 4180.0)	ns

All numerical variables are non‐normally distributed, described by median with the minimum and maximum. Mann Whitney U test were performed to determine p‐value. Categorical variables are described using numbers and percentages. Fisher's exact test were performed to determine p‐value, ns stands for no significant difference.

*Abbreviations*: BMI, body mass index; OD, oocyte donation; IVF, in vitro fertilization.

### 3.2 The extent of macrophage positive surface staining in the decidua

The expression of CD14 and CD163 in the decidua basalis and parietalis was analyzed and compared quantitatively and semi‐quantitatively between the OD group and non‐OD IVF group.

First, the reproducibility of semi‐quantitative and quantitative analyses of the extent of positive staining was evaluated. High correlations were found for intra‐observer variation and inter‐observer variation. High similarity was also shown between semi‐quantitative and quantitative results (Supplementary Material 2).

**FIGURE 1:**
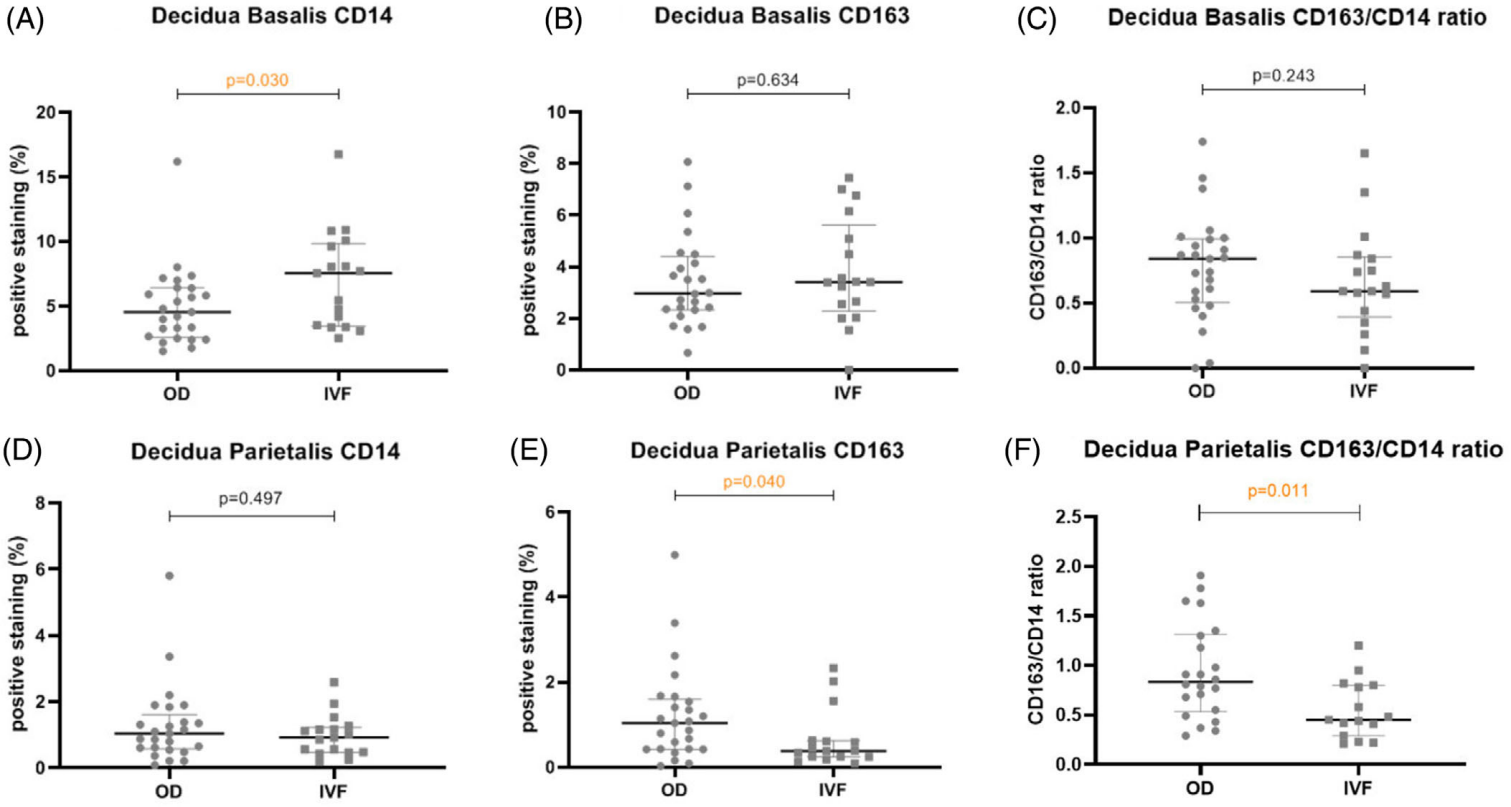
Percentage of positive staining in the decidua basalis and parietalis of CD14, CD 163 and the ratio of CD163/CD14, analyzed quantitatively. The middle horizonal line indicates the median and the whiskers indicate the upper and lower quartiles of the data. Mann‐Whitney U test was performed to identify differences between two groups and p values are shown on the top of the plot

A significantly lower extent of CD14+ staining in basalis was observed in the OD group (median 4.55%) compared to the non‐OD IVF group (median 7.55%) (*P* = .030; Figure 1A). Semi‐quantitative scoring of CD14+ staining gave similar results (Figure S3A). The extent of CD163+ staining in the basalis was not different between groups. The CD163/CD14 ratio in the OD group (median .84) was higher than in the non‐OD IVF group (median .59), but this difference was not significant (Figure 1B, C).

Next, the surface staining in the decidua parietalis of CD14+ and CD163+, along with the CD163/CD14 ratio, was analyzed. In computerized quantitative analysis, CD163‐positive staining was significantly higher in the OD group (median 1.04%) compared to the non‐OD IVF group (median .39%, *P* = .040), but no significant difference was detected in the extent of CD14 positive staining in the parietalis between groups (Figure 1D, E).

When comparing CD163 and CD14, we found that the intensity of CD14 staining in parietalis was generally lower than CD163 staining in parietalis. Thus, the thresholds for CD14 positive signals were set higher than those for CD163 positive signals, to only measure specific staining signal and avoid including measurement of background staining in the tissue sections. As ratio between extent of CD163 staining and extent of CD14 staining was peculiarly high (> 3) in some cases, we suspected the difference in intensity of the two stainings might be explaining these high ratios. Indeed we found a significant correlation between staining intensity ratio and staining extent ratio (Spearman's r = −.47, R^2 ^= .22, Spearman's *P* = .002). Therefore, four samples with extreme intensity ratios (either < .5 or > 1.5) were excluded from the analyses altogether (see detailed methods in Supplementary Material 4 and results in Figure S4), resulting in disappearance of the significant correlation between staining intensity ratio and staining extent ratio (Figure S4b). After this correction, the ratio of extent of CD163 to CD14 surface staining was significantly higher in the OD group (median .84) compared to the non‐OD IVF group (median .45, *P* = .032) (Figure 1F).

**FIGURE 2:**
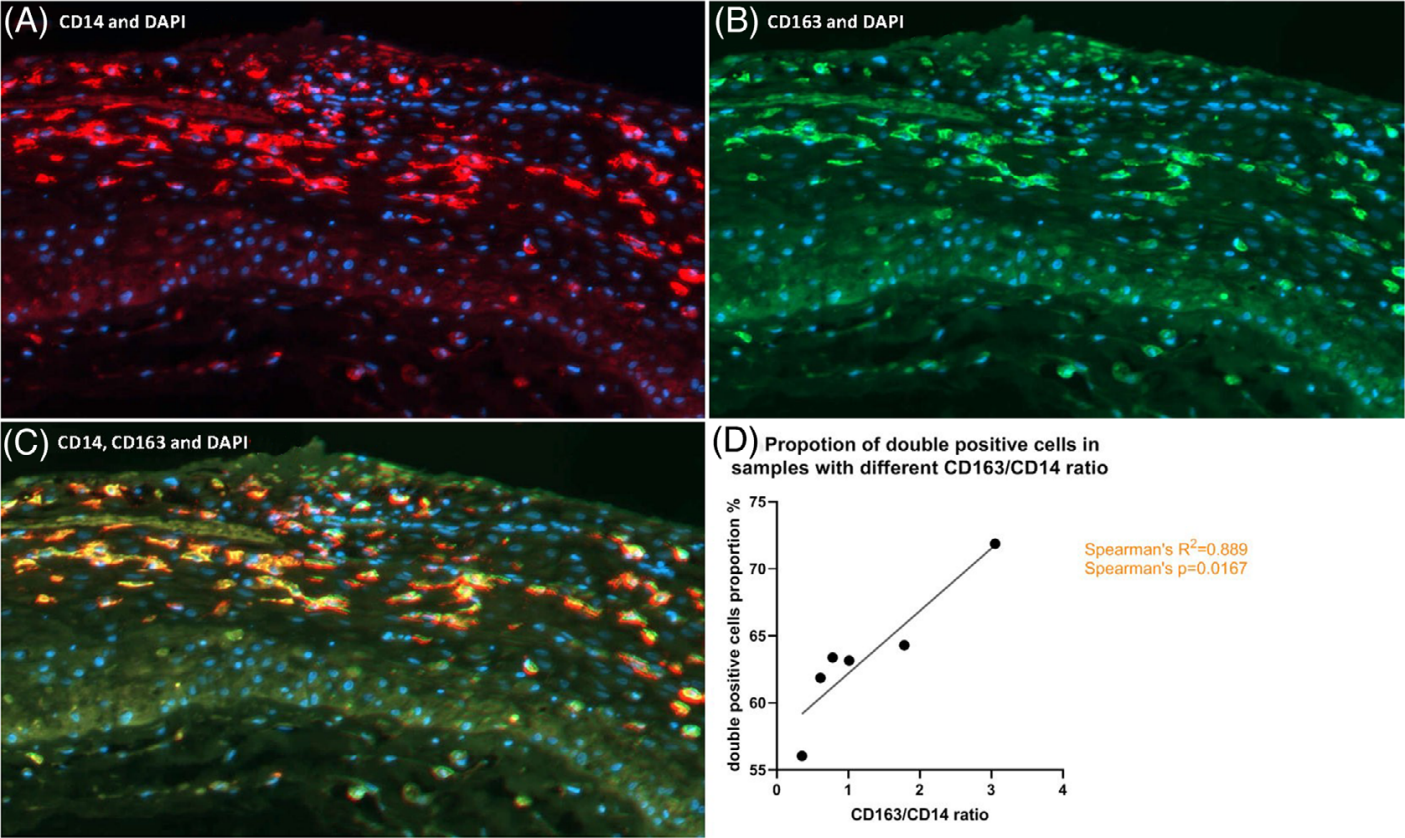
(A‐C). Double immunofluorescence staining of CD14 and CD163. Example of decidua parietalis stained for colocalization (300x magnification). Shown are the nuclei (blue) with single staining for CD14 (red in A), CD163 (green in B), and the combined double staining of CD14 and CD163 (yellow in C). (D). Dot plot shows six samples with different CD163/CD14 ratios in immunohistochemistry surface analysis, and their proportion of CD14/CD163 double positive cells in the total number of CD14‐positive cells in double label immunofluorescence staining. Spearman's correlation was performed to assess the correlation between the analyses

We wanted to test if the CD163/CD14 ratios measured in the decidua reflect the cells that are double positive for CD14 and CD163. Therefore, a double label immunofluorescence staining was performed for three decidua basalis and three decidua parietalis samples, showing either a high, medium or low ratio. In total, 4255 single or double CD14 positive cells were analyzed. The double staining confirmed that part of the cells being positive for the general macrophage marker also showed positivity for CD163 (Figure 2A‐C). In the total sample set, the immunohistochemical CD163/CD14 ratio highly correlated with the proportion of CD163/CD14 double‐stained cells in immunofluorescence (Spearman's R^2 ^= .89, Spearman's *P* = .017; Figure 2D).

Since the sampling time has significant difference between the two groups (*P* = .028) and the median of the sampling time in the OD group is higher than that in the non‐OD IVF group, we performed the correlation analysis (Spearman's correlation) between outcomes and sampling time within the groups: none of them showed significant correlation. Although the comparison of clinical parameters showed no significant differences between the two groups, we still performed the correlation analysis between the outcomes (CD14, CD163 and CD163/CD14 ratio in decidua basalis and parietalis) and patient characteristics (maternal age and gestation age), which all showed no significant correlation (Spearman's correlation).

### 3.3 Association between the ratio of CD163/CD14 positive staining and fetal‐maternal HLA mismatches

We wanted to test whether differences in macrophage composition are related to fetal‐maternal HLA incompatibility. Therefore, we divided the OD cohort into a semi‐allogeneic group (≤5 fetal‐maternal HLA mismatches) and a fully allogeneic group (> 5 fetal‐maternal HLA mismatches). Clinical data of the three groups, namely non‐OD IVF group, semi‐allogeneic OD group and fully allogeneic OD group, showed no significant differences (data not shown).

**FIGURE 3:**
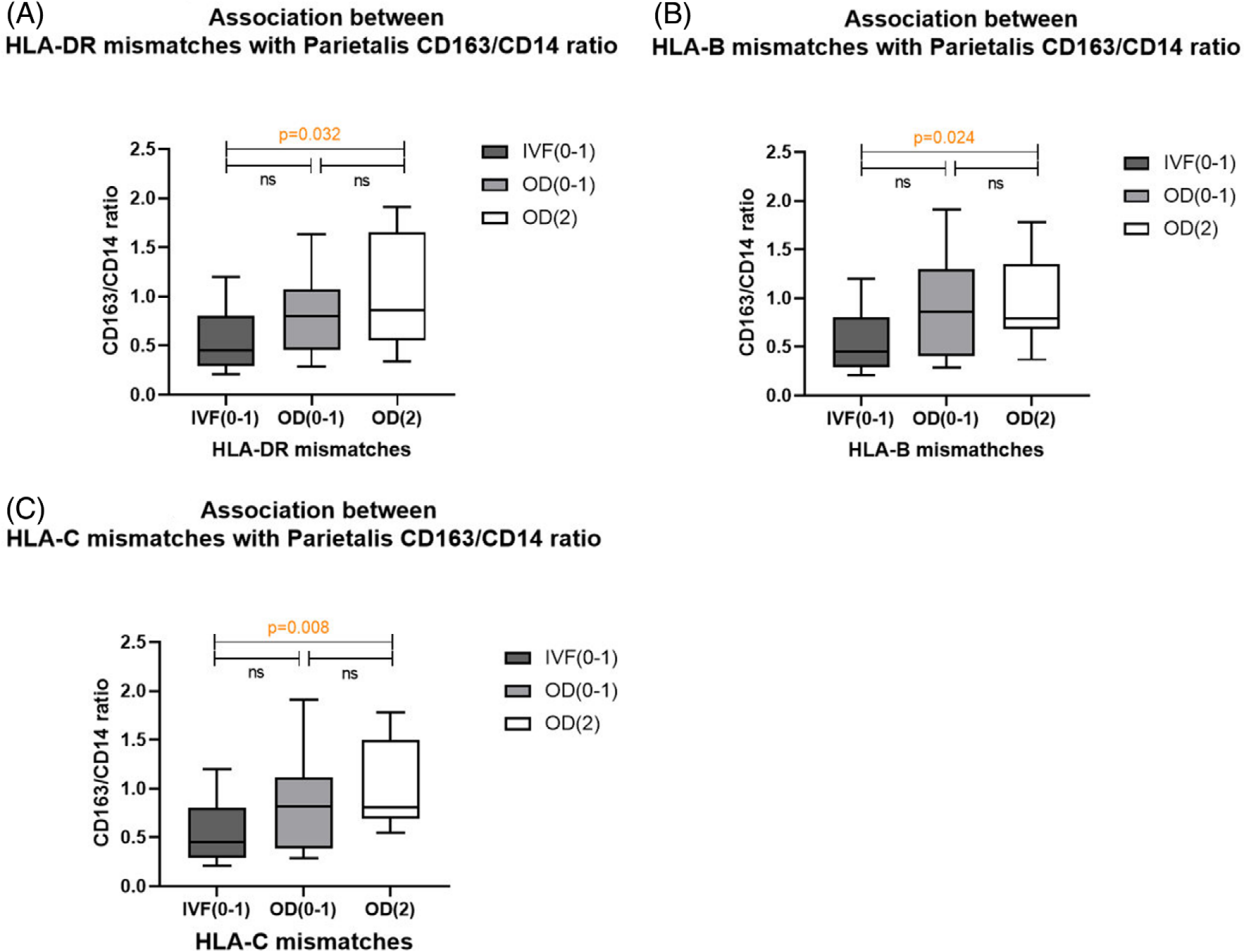
Association of ratio of CD163/CD14 and CD163 positive staining in decidua parietalis with the extent of HLA mismatching. The numbers in parentheses indicate the range of HLA mismatches for each group: IVF (0‐1) refers to non‐OD IVF group, OD (0‐1) refers to semi‐allogeneic OD group, OD (2) refers to fully allogeneic OD group. The middle horizonal line within the box indicates the median, the ends of the box correspond to the upper and lower quartiles of the data, and the whiskers indicate minimum and maximum values. Mann‐Whitney U tests were performed to identify differences between two groups. Significant differences are shown with p value, ns means non‐significant

Since the CD163/CD14 ratio showed significant difference between OD and non‐OD IVF group in decidua parietalis, but not in decidua basalis, here we concentrate on the association between the CD163/CD14 ratio in decidua parietalis and fetal‐maternal HLA mismatching. First, at individual locus level, we found that the HLA‐DR‐fully‐allogeneic OD group showed a higher CD163/CD14 ratio than the non‐OD IVF group (*P* = .047) (Figure 3A). This significantly higher CD163/CD14 ratio was also found when comparing HLA‐class‐II‐fully‐allogeneic OD pregnancies with non‐OD IVF pregnancies (*P* = .047) (Figure S5A).

There was also a significantly higher CD163/CD14 ratio in the HLA‐B‐fully‐allogeneic and HLA‐C‐fully‐allogeneic OD group than in the non‐OD IVF group (*P* = .037 and *P* = .027, respectively) (Figure 3B, C). In line with this finding, a significantly higher percentage of CD163 positive staining in decidua parietalis was observed when comparing HLA‐B‐ or HLA‐C‐fully‐allogeneic OD group with non‐OD IVF group (*P* = .010 and *P* = .023, respectively) (Figure S5B, C). This significantly higher CD163 positive staining was also found in the HLA‐class‐I‐fully‐allogeneic OD group compared to non‐OD IVF group (*P* = .047) (Figure S5D).

In addition, when comparing total‐HLA (class I+II)‐fully‐allogeneic group with non‐OD IVF group, a significantly higher CD163/CD14 ratio and CD163 positive staining were observed (*P* = .019 and *P* = .031, respectively) (Figure S5E, F).

## 4 DISCUSSION

We found in the decidua parietalis that the percentage of CD163 positive cells and the CD163/CD14 ratio were significantly higher in the OD group compared to the non‐OD IVF group. In decidua basalis, there was a significantly lower percentage of CD14 positive cells in the OD group compared to the non‐OD IVF group. Consequently, the CD163/CD14 ratio was higher in the OD group than in the non‐OD IVF group, although this difference did not reach significance. Our study also suggests the association between the CD163/CD14 ratio and fetal‐maternal HLA mismatching, especially for the HLA‐DR, ‐B, and ‐C loci.

Our findings are in line with an article from Nakabayashi et al., where they also reported a significant decrease of total macrophages in the decidua basalis in normotensive women of OD pregnancies when compared to normotensive women of naturally conceived or IVF pregnancies,^25^ though they used CD68 as marker for macrophages instead of CD14. Both of these markers are overtly present on monocytes and are abundantly expressed by macrophages.^26^, ^27^ CD14 is anchored on the surface of the cell membrane,^26^ while CD68 is mainly located in the cytoplasm but can rapidly shuttle to the cell surface.^27^ Another study established by Schonkeren et al. reported a special lesion in the chorionic plate of OD pregnancies, where CD14 and CD163 macrophages markers are significantly upregulated.^28^ Nakabayashi hypothesized that the decrease of macrophages in the decidua basalis and the increase of macrophages in the chorionic plate might be due to migration of macrophages from one layer to the other.^25^

However, both these studies did not calculate the percentage of CD163 positive macrophages (M2 macrophages) in the total number of macrophages, which is the main finding of our study. CD163 has been suggested as a marker of anti‐inflammatory macrophages,^13^, ^18^ and CD14 is considered to be present on all macrophages, irrespective whether they are pro‐inflammatory or anti‐inflammatory. Thus the CD163/CD14 ratio was calculated to determine the polarization of the decidual macrophages. Since the chorionic plate, villi, and basal plate are all directly in contact with maternal tissues or maternal blood in the intervillous space, fetal‐maternal interactions may be occurring at each of these layers.^29^ Thus, immune responses would be expected to achieve fetal‐maternal tolerance in all these locations. Therefore, besides the migration of immune cells from one place to the other, the polarization of macrophages might be an explanation for the changes of general and M2 macrophage markers in different layers of the placenta. As all OD pregnancies included in our study were maintained until term delivery without complications, the higher ratio of decidual M2 macrophages, which are generally believed to have anti‐inflammatory and immunoregulatory effect,^19^, ^30^ may reflect a local compensatory mechanism to inflammatory conditions to make sure that the fetus is retained until the end of pregnancy. Although decidual macrophages were reported to mediate a pro‐inflammatory environment at the very beginning of implantation^31^ and at term,^32^ this argument does not refute our hypothesis as the sampling time between the two study groups was similar. Admittedly, in this study we only used CD163 as the surface marker for M2 macrophages, and the exact functional role of these CD163 positive cells needs to be further defined. It is reported that a high CD163 expression in macrophages is a characteristic of anti‐inflammatory responses, accounted to the potential to scavenge cellular debris, produce anti‐inflammatory cytokines such as IL‐10, and limit progression of inflammatory reactions.^33^, ^34^ These functions can be induced by trophoblast cells and might be cooperating with other immune suppressive cells like T regulatory cells.^12^

While the villous trophoblast cells invade into decidua basalis but not decidua parietalis, our study found that the significance of outcomes of the CD163/CD14 ratio was more prominent in the decidua parietalis. A larger fetal‐maternal interacting surface area in decidua parietalis than in decidua basalis might be an explanation. Our finding is in line with another study of mass cytometry on term decidua of healthy pregnancies established by Van der Zwan et al, where they also showed a significantly higher proportion of CD163+CD14+ cells in the decidua parietalis compared to the decidua basalis.^35^ In addition, a higher proportion of regulatory T cells among CD4+ T cells in decidua parietalis compared to decidua basalis^36^ might be related to the phenomenon of macrophages in the current study. Our study only focused on decidua basalis and parietalis. Future studies could further sort out the polarization of macrophages at the chorionic plate and other locations of the placenta. Meanwhile, it is also worthwhile to explore whether the macrophages are from maternal or fetal origin in each placental layer.

Since our immunohistochemistry results are measured in pixels, which are not equal to individual cells, we further performed double immunofluorescence staining and cell segmentation on several samples with various CD163/CD14 ratios to test whether cells were double positive for CD14 and CD163. According to the positive correlation between CD163/CD14 ratio in immunohistochemistry staining and the proportion of double positively labeled cells in immunofluorescence staining, the accuracy of using positive surface staining to evaluate cell quantity was confirmed.

It is expected that in the double staining all CD163+ cells are positive for CD14+ and that consequently the CD163/CD14 ratio is lower than 1, as CD14 is present on all macrophages and CD163 is only present on a subset of anti‐inflammatory macrophages.^10^, ^13^ Yet, several samples in our study had a ratio higher than 1 and a few single CD163 positive cells were also observed, which might suggest that not all CD163+ cells are CD14+. An earlier study of patients with preeclampsia and preterm birth found a unique subset of CD14−/CD68+ cells in the placenta, and concluded that these cells were a subpopulation of macrophages based on their immunophenotypic characteristics.^37^ However, another study found a CD14‐CD163+ dendritic cell subset, secreting intermediate quantities of pro‐inflammatory mediators.^38^ The presence of a CD14‐ group is hinted to but not proven in our study. Future study with larger sample size might be more suitable to identify a new subset in placenta. Moreover, functional tests will be needed to determine the characteristics of such subset. An alternative explanation of some samples exceeding 1 or even 2 might be that the CD14 staining was less intense than the CD163 staining, which became more apparent in the parietalis sections. Although both stainings worked optimal and were applied at the same time on the whole series of slides from both study groups, elevating the threshold for detecting CD14 specific signal may have led to loss of weak but specific CD14 signal in some samples.

The association between macrophages and HLA mismatches was analyzed as well. For evaluating associations we concentrated on CD163 positive staining and CD163/CD14 ratio in decidua parietalis, since they showed the largest differences between groups. To eliminate confounding variables and to only focus on HLA mismatches, we separated the OD group into a semi‐allogeneic and a fully allogeneic group. Clinical data of these two groups and the non‐OD IVF group showed no significant differences. Therefore, only the number of HLA mismatches was the independent variable between semi‐allogeneic and fully allogeneic OD group. Unfortunately, between the semi‐allogeneic and fully allogeneic OD group no significant difference was found for CD163 positive staining or CD14/CD163 ratio in the decidua parietalis, possibly due to the limited sample size in our study. However, when comparing fully allogeneic OD group with non‐OD IVF group, a significantly higher percentage of CD163 positive cells and higher CD163/CD14 ratio were observed in the decidual parietalis. These differences were not found when comparing semi‐allogeneic OD group with non‐OD IVF group, suggesting that the extent of HLA mismatching between mother and fetus remains a possible factor associated with the quantity and the composition of macrophages in the placenta of OD pregnancies. A significant correlation between HLA mismatches and macrophages in OD pregnancies might be found if sample size would be enlarged.

Among all the five HLA loci that we calculated in our study, HLA‐DR incompatibility was related to the most prominent difference in macrophage composition between groups. We found that the association not only exist between macrophages composition and HLA‐DR mismatches, but continue to exist with HLA class II, which includes HLA‐DR. Though limited types of HLA molecules are detected on the trophoblast, among which HLA‐C is the only classical HLA antigen, other studies pointed to a possible pathophysiologic role of HLA‐DR in pregnancies. Tersigni et al. demonstrated that HLA‐DR can be detected on syncytiotrophoblasts from placentas of women with preeclampsia.^39^ Moreover, van Bentem et al. showed that mismatching at HLA class II, including HLA‐DR, independently was associated with the development of preeclampsia in OD pregnancies.^40^ In maternal peripheral blood of uncomplicated OD pregnancies, van der Hoorn et al. found a positive correlation between the number of HLA‐DR mismatches with the number of CD4+CD25^dim^ cells.^41^ Thus, HLA‐DR is worthy considering in OD pregnancy, as HLA‐DR mismatches might have effect on modifying the immune environment during pregnancy by changing the quantities of certain immune cells, including macrophages in placenta. In addition, we also found association between HLA‐B, ‐C mismatches and the CD163/CD14 ratio in the decidua parietalis.

The association between decidual macrophages and HLA antibodies, which can be induced during pregnancy, is also worthy for further investigation. The levels of HLA antibodies in women with OD pregnancies have been studied: Lashley et al. found that women with OD pregnancies have a higher chance of developing child‐specific HLA antibodies, and interestingly that this high incidence of HLA class I antibody production strongly depends on the extent of fetal‐maternal HLA‐DR incompatibility.^42^ However, while HLA antigen mismatches are observed to be associated with the development of preeclampsia in OD pregnancies, the influence of HLA antibodies on development of preeclampsia is uncertain.^40^

The exact mechanism of how macrophages would respond to larger genetic dissimilarity is not yet clear and needs to be further established in the future. Macrophages possess abundant functions, including their interaction with and effect on other immune cells. For example, they release cytokines and enzymes to reduce the cytolytic killing of trophoblasts by decidual natural killer (NK) cells, limit T‐cell activation, and induce regulatory T cells.^14^ It is possible that a higher extent of fetal‐maternal HLA mismatching has direct effects on decidual NK cells and T cells,^43^, ^44^ and then macrophages may act as regulators of immune modulation in such an immune micro‐environment. Another probability is based on the remodeling function of macrophages. The placentation, which includes the invasion of extravillous trophoblast and the remodeling of spiral arterials, might become impaired and abnormal when fetal‐maternal genetic dissimilarity is larger. But macrophages gathering around the spiral arterials might be able to compensate for this impairment and dissimilarity.^13^

In conclusion, we found a higher extent of CD163 positive M2 macrophages within the total decidual macrophage load of uncomplicated OD pregnancy than in non‐OD IVF pregnancies. The observations may demonstrate that a compensatory mechanism is needed in order to deal with the higher HLA incompatibility between fetus and mother in OD pregnancies. The exact mechanism is yet to be established.

## CONFLICT OF INTEREST

All authors have no conflicts of interest to declare relevant to this study.

## Supporting information

Supporting Information

Supporting Information

Supporting Information

Supporting Information

Supporting Information

Supporting Information

## Data Availability

The authors confirm that the data supporting the findings of this study are available within the article and its supplementary materials.
