# Supplementary material for: Uncomplicated oocyte donation pregnancies display an elevated CD163‐positive type 2 macrophage load in the decidua, which is associated with fetal‐maternal HLA mismatches
Source: Am J Reprod Immunol. 2021 Dec 4;87(1):e13511. doi: 10.1111/aji.13511 (PMC9286476; doi:10.1111/aji.13511)
Supplement: Supplementary file 1 — Supporting Information [file AJI-87-0-s004.docx]

**Supplementary Material:**

**Supplementary Material** **1:**


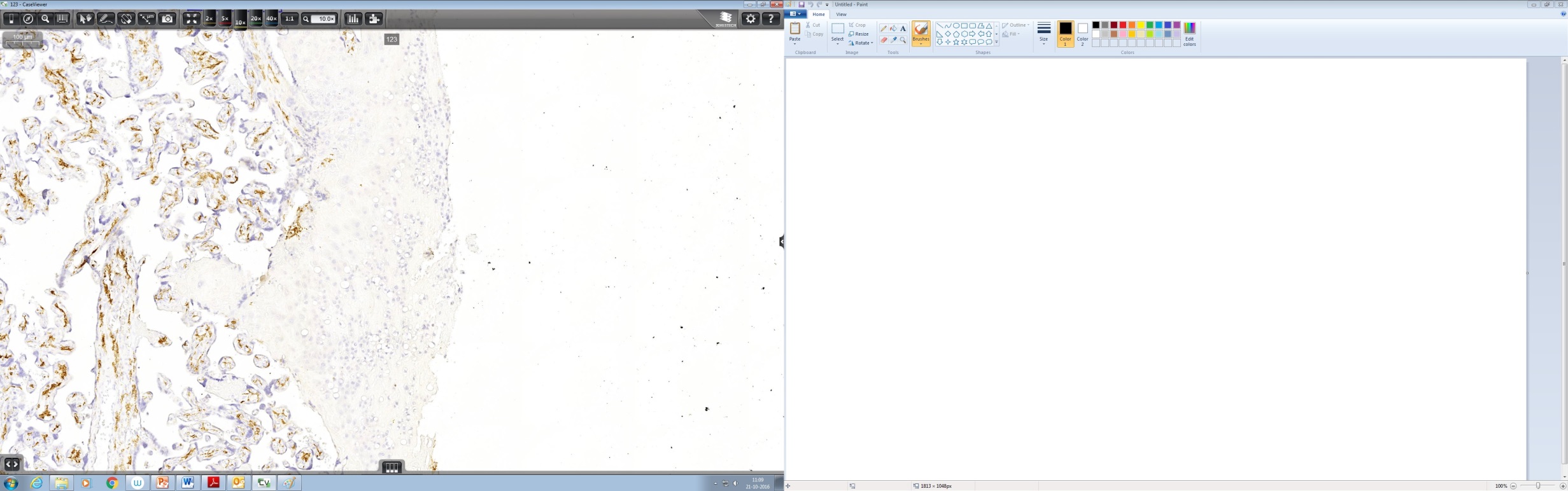

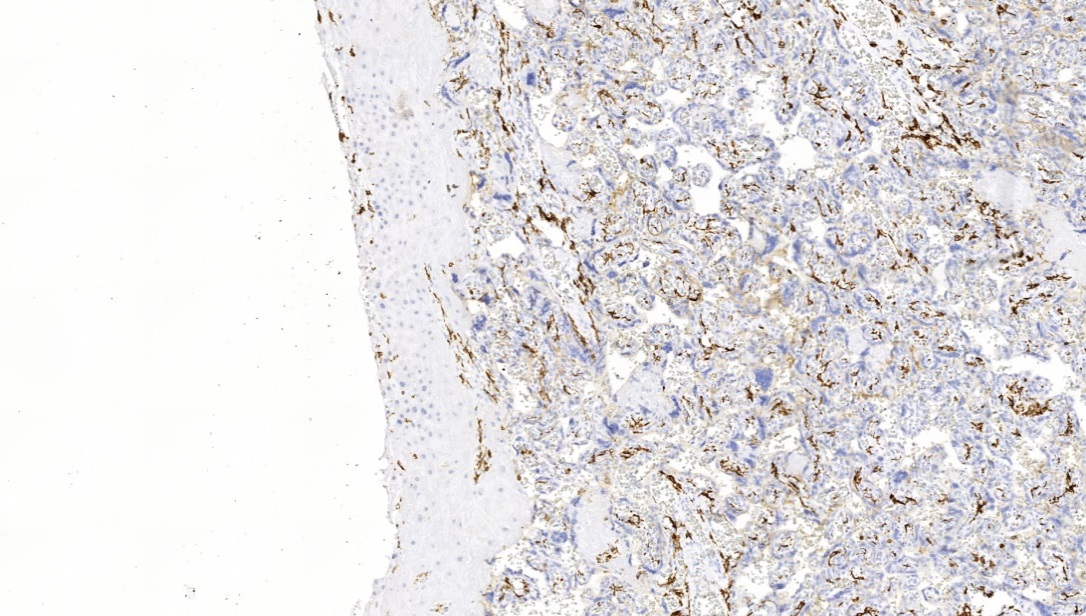

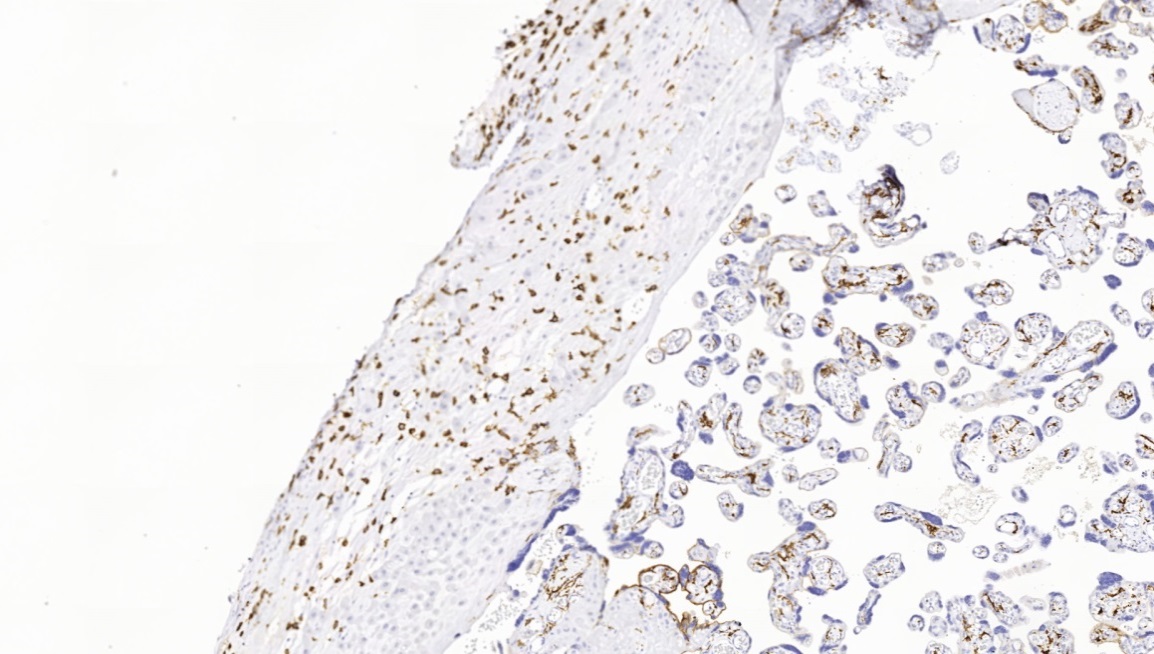

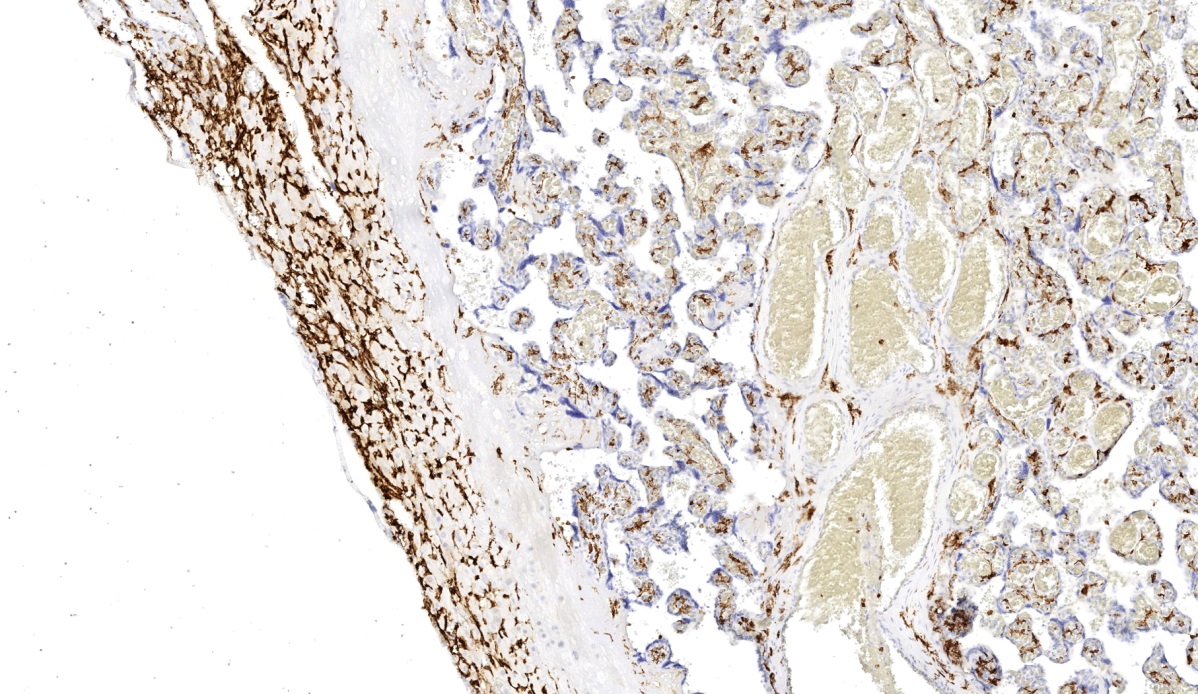


**D**

**C**

**B**

**A**


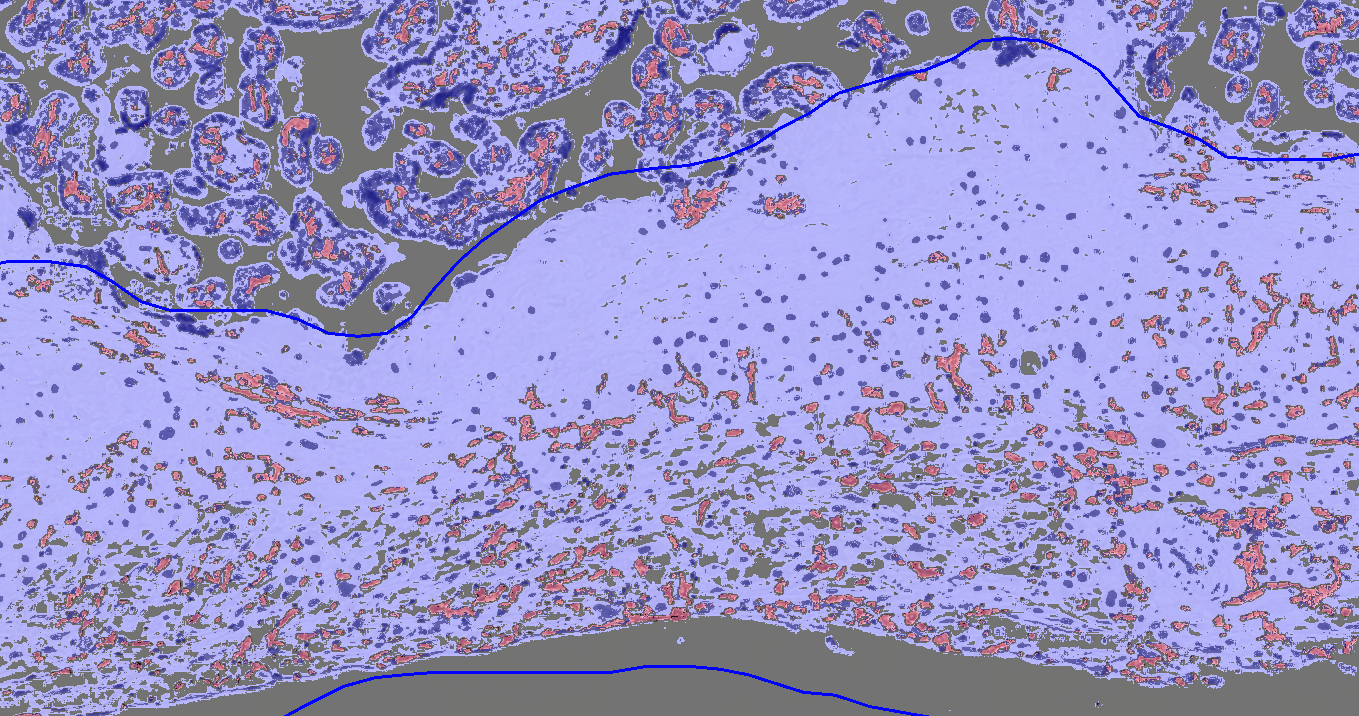

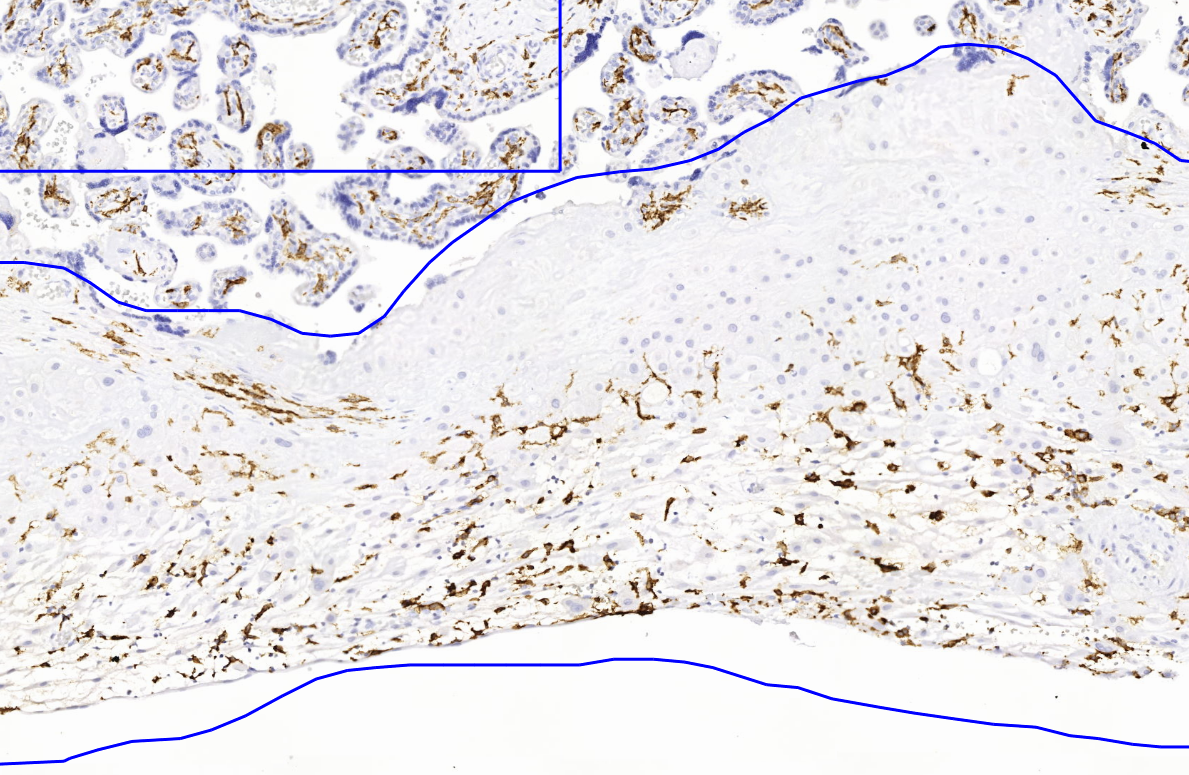


**F**

**E**

**E**

**Supplementary figure 1.** Scoring of the decidua (with CD163 staining). A-D Semi-quantitative scoring of the decidua basalis. E-F. Quantitative training of Quant studio software.

**F**

**
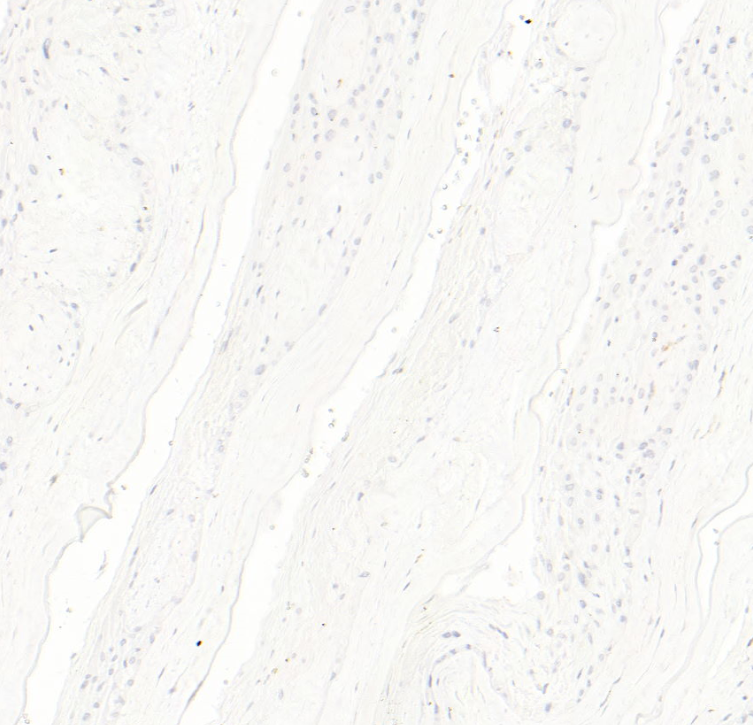

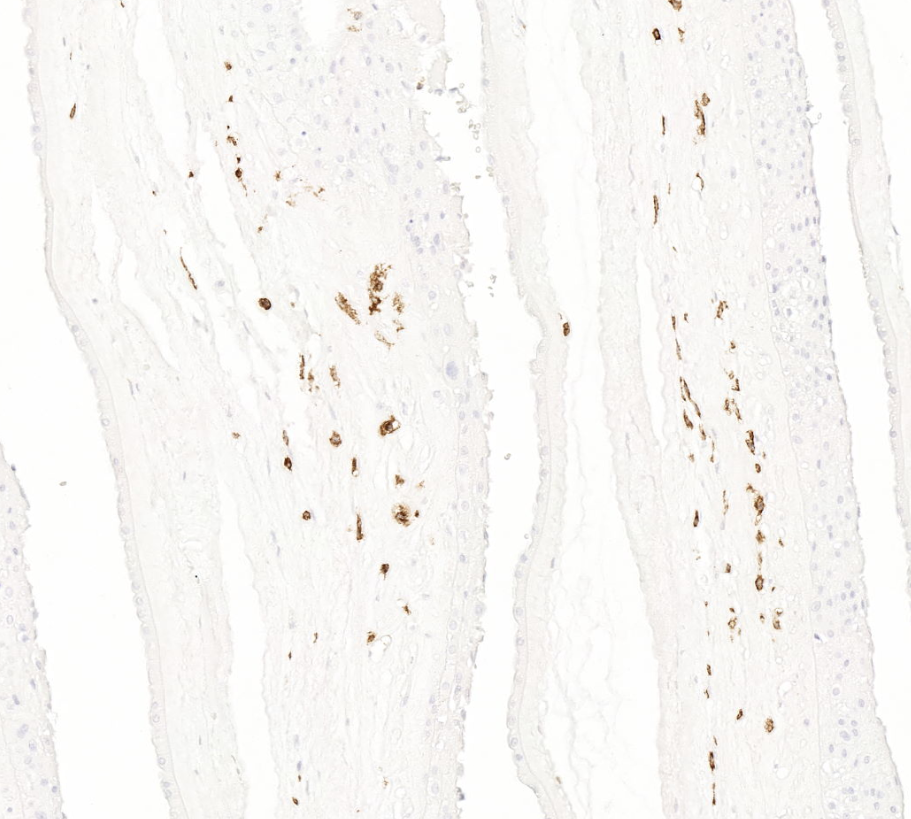

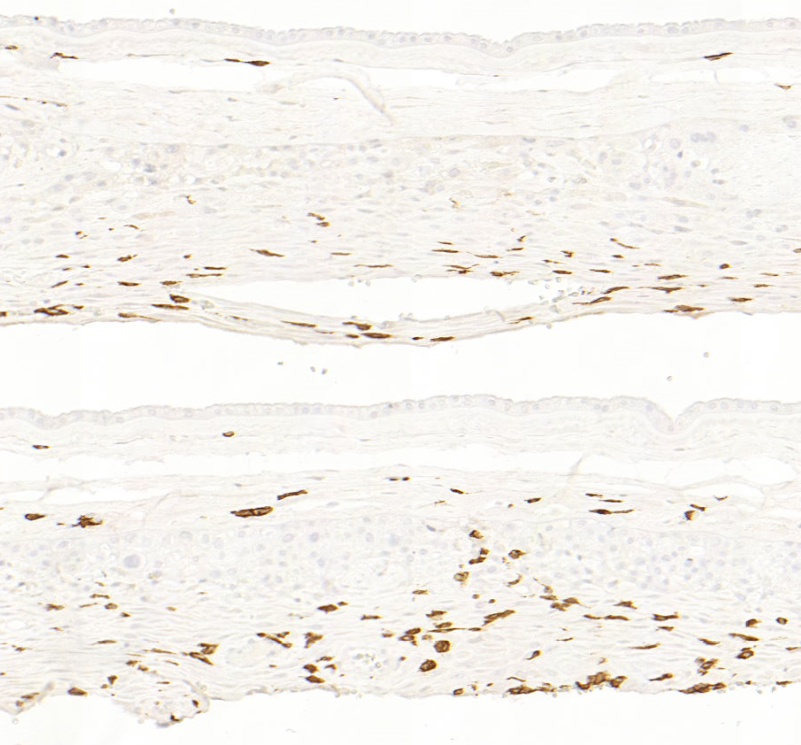

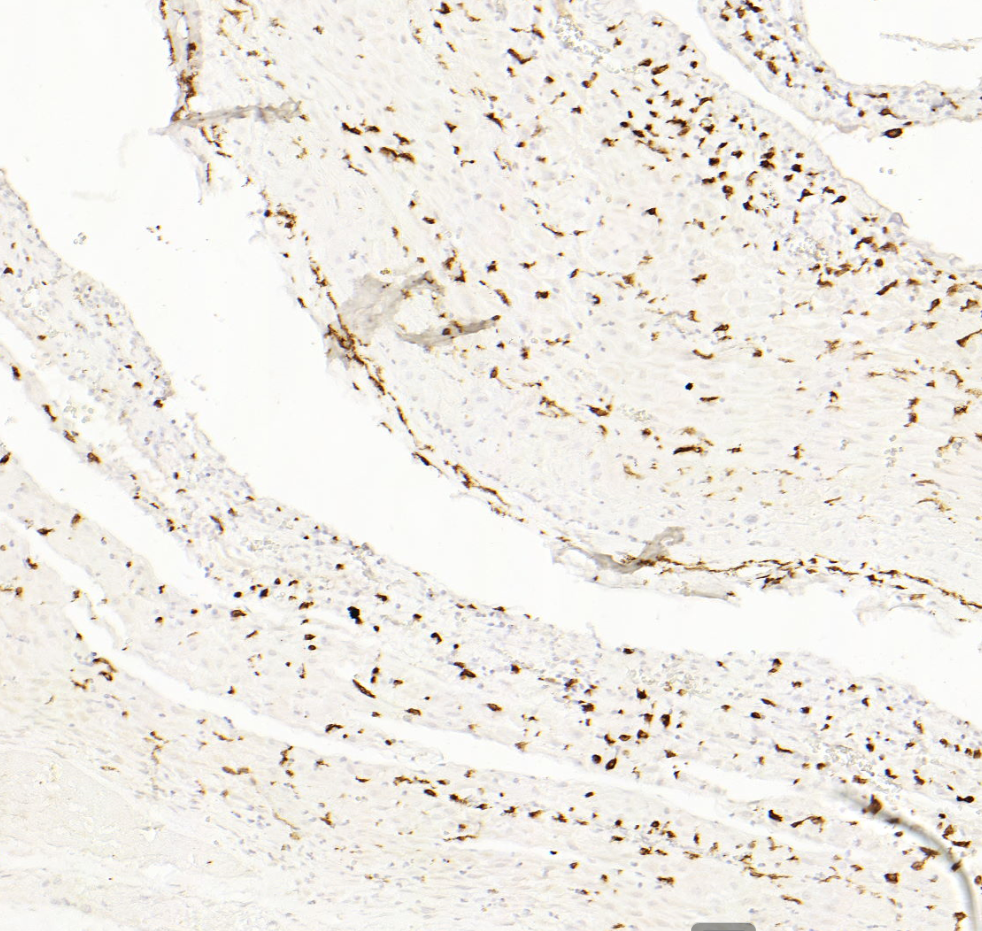

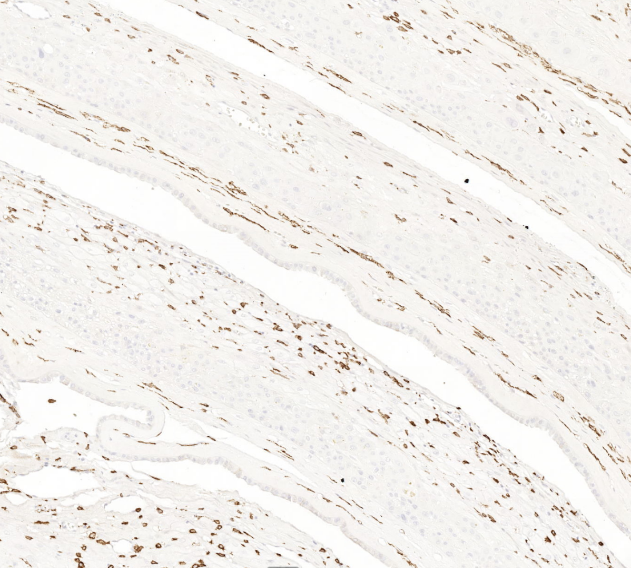

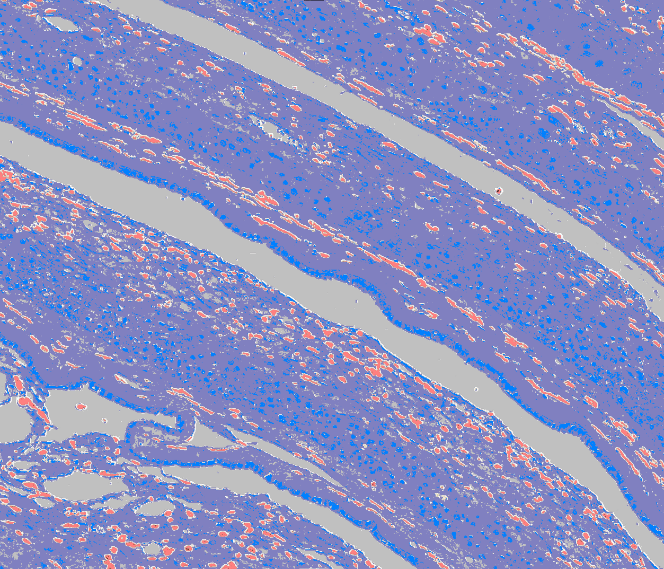
**

**F**

**E**

**D**

**C**

**B**

**A**

**Supplementary figure 2.** Scoring of the parietalis (with CD163 staining). A-D Semi-quantitative scoring of the decidua parietalis. E-F. Quantitative training of Quant studio software.

**Supplementary Material** **2**: Consistency between two investigators and consistency between quantitative and semi-quantitative scoring.

Fifteen basalis slides and twenty parietalis slides were randomly selected and scored by one observer at different time points to determine intra-observer variability. The reproducibility was high in basalis (semi-quantitatively: Cohens Kappa= 1.00 (perfect); quantitatively: Spearman r^2^ = 0.97 (very high correlation)) and in parietalis (semi-quantitatively: Cohens Kappa= 1.000 (perfect); quantitatively: Spearman r^2^ = 0.93 (very high correlation)).

The same slides were scored by two observers to assess the inter-observer variability. Substantial agreement was achieved both in basalis (semi-quantitatively: Cohen’s Kappa = 0.67 (good); quantitatively: Spearman r^2^ = 0.52 (high correlation)) and parietalis (semi-quantitatively: Cohen’s Kappa = 0.83 (excellent); quantitatively: Spearman r^2^ = 0.99 (very high correlation)).

The correlation between semi-quantitative and quantitative results were tested to evaluate the similarity of the approaches. The correlation was high for both basalis (Spearman r^2^ = 0.53) and parietalis (Spearman r^2^ = 0.78 (high correlation)).

**Supplementary Material 3:** Semi-quantitative scoring of CD14 and CD163 surface staining in the decidua


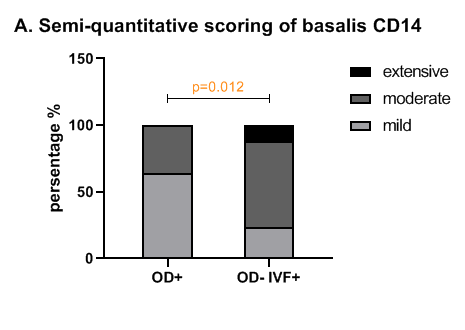

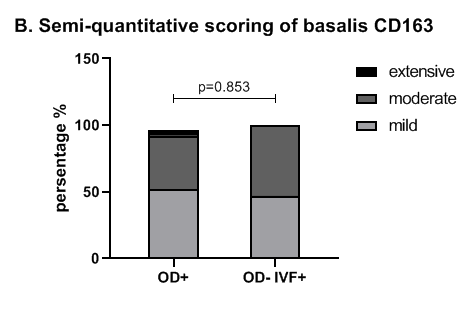


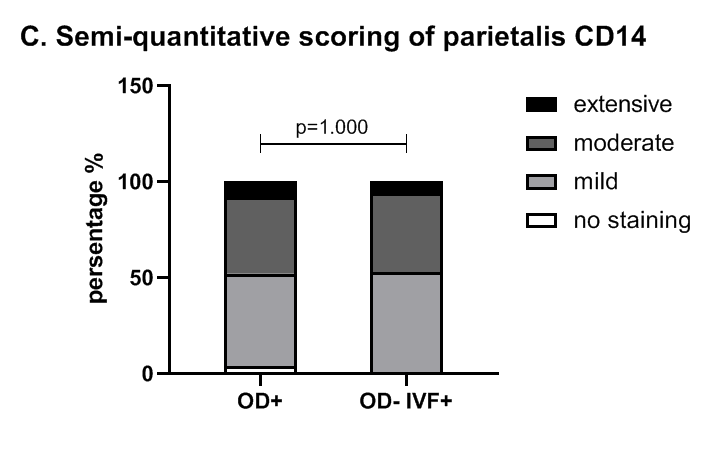

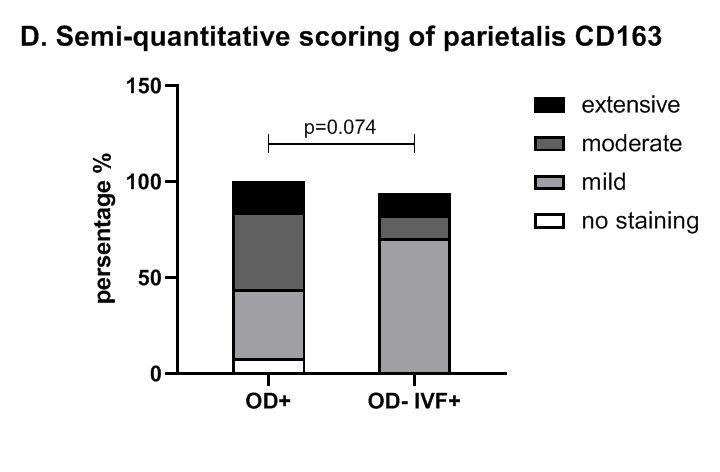


**Supplementary Figure 3:** Semi-quantitative scoring of the basalis and parietalis. Percentage of each staining density was shown in histogram. Fisher’s exact test was performed to identify differences between two groups and p value was shown on the top of the histogram.

**Supplementary Material 4:** CD14 and CD163 staining intensity analysis in decidua parietalis.

**Methods:** The scoring was performed at a 10X zoom on a 24-inch screen. 10 cells were randomly chose on both CD14 and CD163 stained slides of each sample. The values of three primary colors (red, green, blue; RGB) of the darkest part in each cell were measured (The lower the RGB values were, the darker the staining color was). Then the average RGB values of those 10 cells were calculated as the intensity values of CD14 and CD163 staining respectively. After calculating the ratios of these intensity values (CD163/CD14), the correlation between these intensity ratios and the positive surface staining ratios was analyzed.

**Figures:**


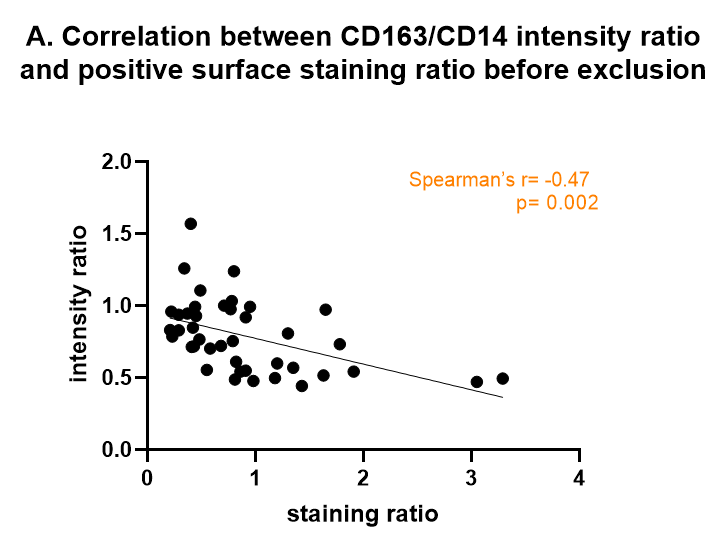

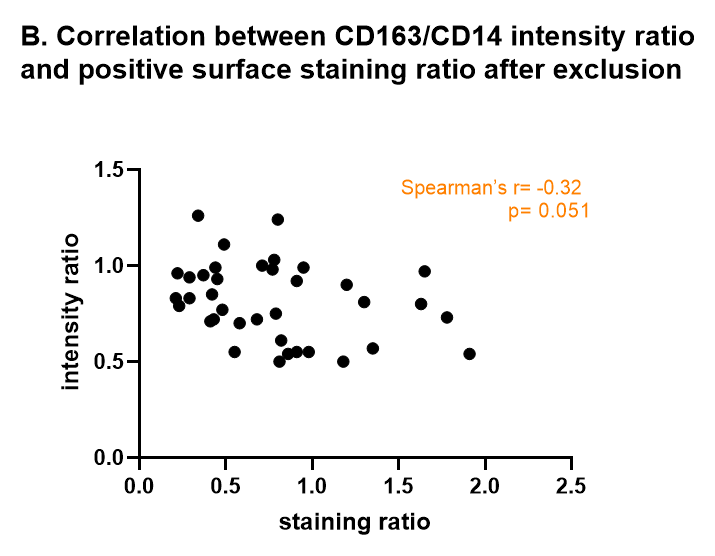


**Supplementary Figure 4:** Correlation between CD163/CD14 intensity ratio and positive surface staining ratio, before and after excluding 4 outliers (intensity ratio <0.5 and >1.5). Significant negative correlation is found before exclusion, but changed into non-significant after exclusion. P value and r of Spearman’s correlation are shown on the figures.

**Supplementary Material 5:** Association between ratio of CD163/CD14 and CD163 positive staining in decidua parietalis and the extent of HLA mismatching.


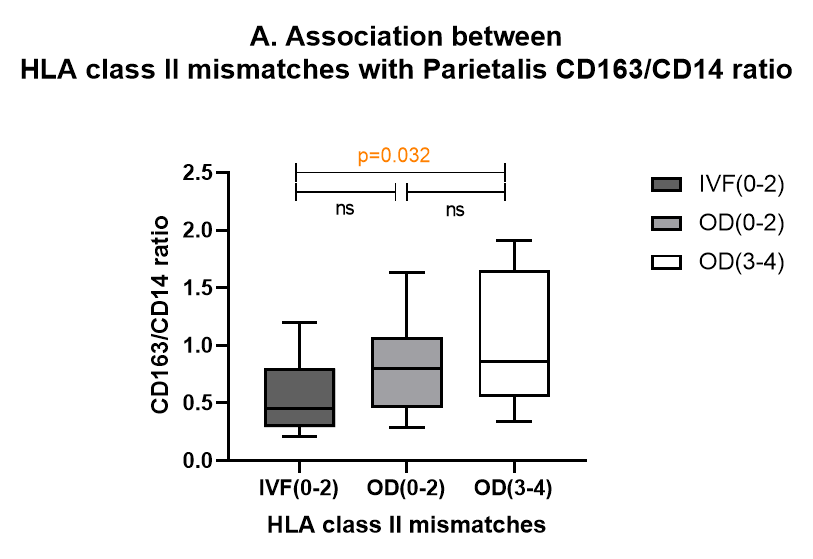

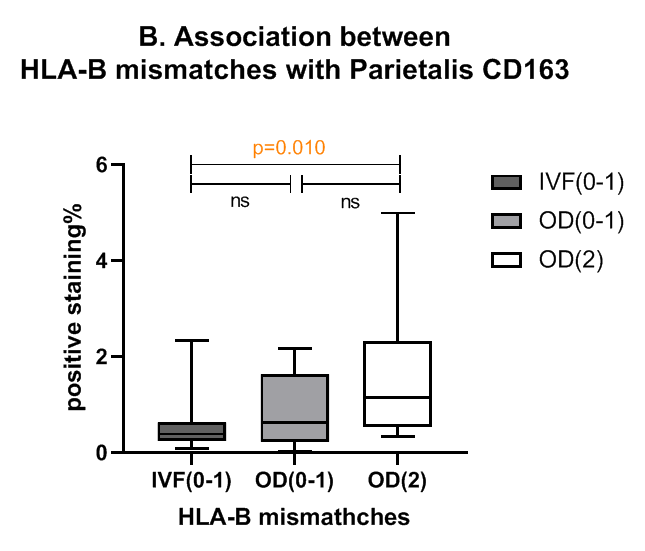


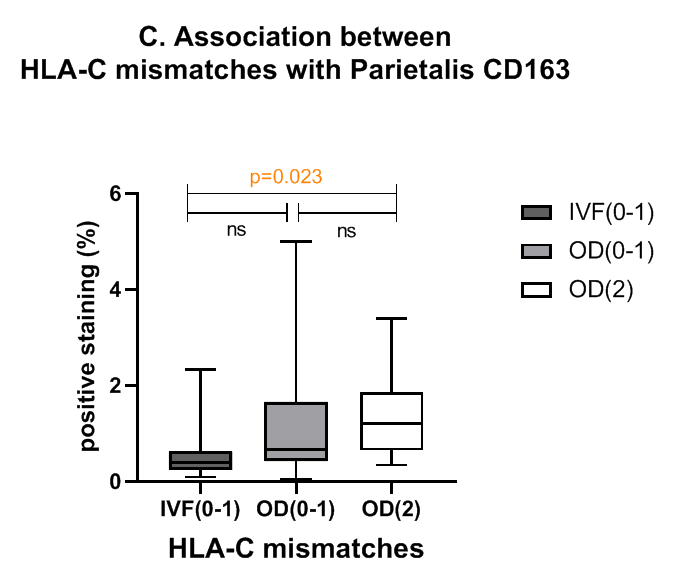

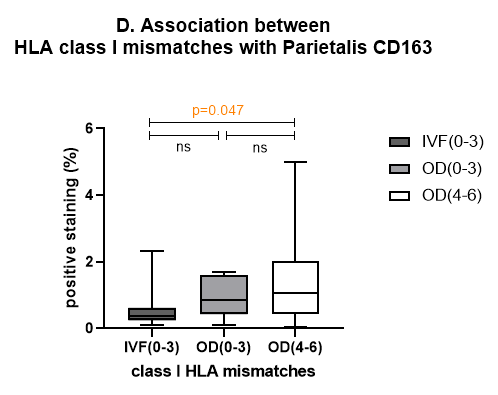


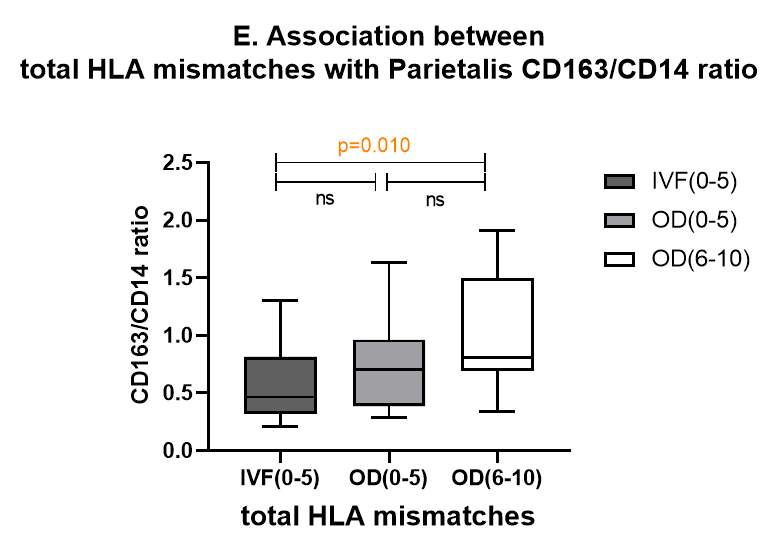

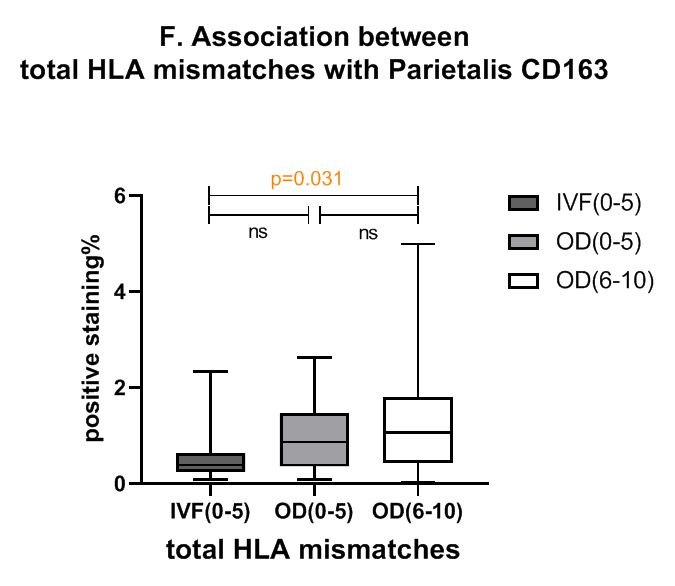


**Supplementary Figure 5:** Association of ratio of CD163/CD14 and CD163 positive staining in decidua parietalis with the amount of HLA mismatching. The numbers in parentheses indicate the range of HLA mismatches for each group, IVF(0-1), IVF(0-2), IVF(0-3) and IVF(0-5) refer to non-OD IVF group; OD (0-1), OD (0-2), OD (0-3) and OD (0-5) refer to semi-allogeneic OD group; OD (2), OD (3-4), OD (4-6) and OD (6-10) refer fully allogeneic OD group. The middle horizonal line within the box indicates the median, the ends of the box correspond to the upper and lower quartiles of the data, and the whiskers indicate minimum and maximum values. Mann-Whitney U tests were performed to identify differences between two groups. Significant differences are shown with p value, ns means non-significant.
