# Supplementary figures and images for: Uncomplicated oocyte donation pregnancies display an elevated CD163‐positive type 2 macrophage load in the decidua, which is associated with fetal‐maternal HLA mismatches
Source: Am J Reprod Immunol. 2021 Dec 4;87(1):e13511. doi: 10.1111/aji.13511 (PMC9286476; doi:10.1111/aji.13511)

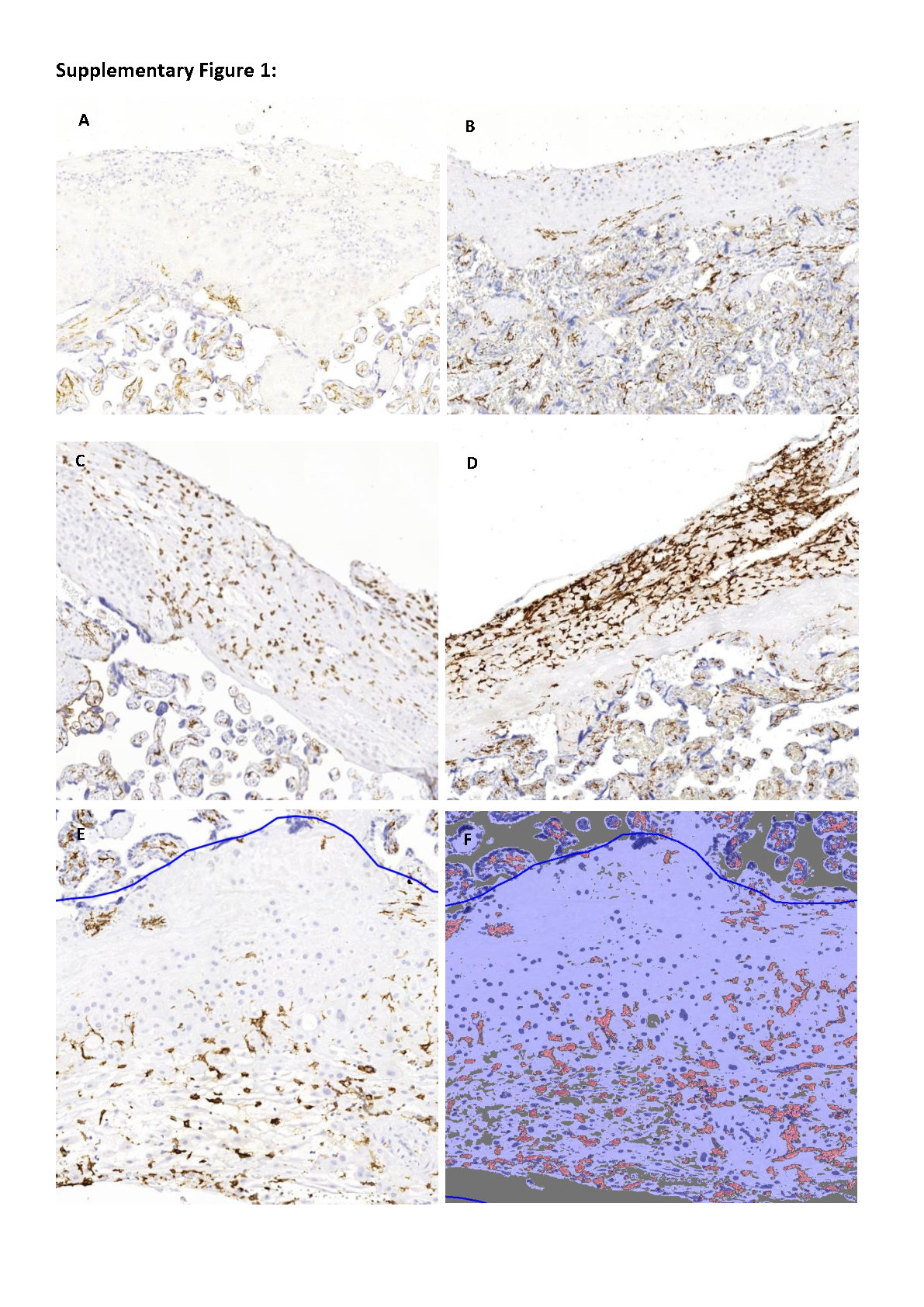

Supplement: Supplementary file 2 — Supporting Information [file AJI-87-0-s003.jpg]

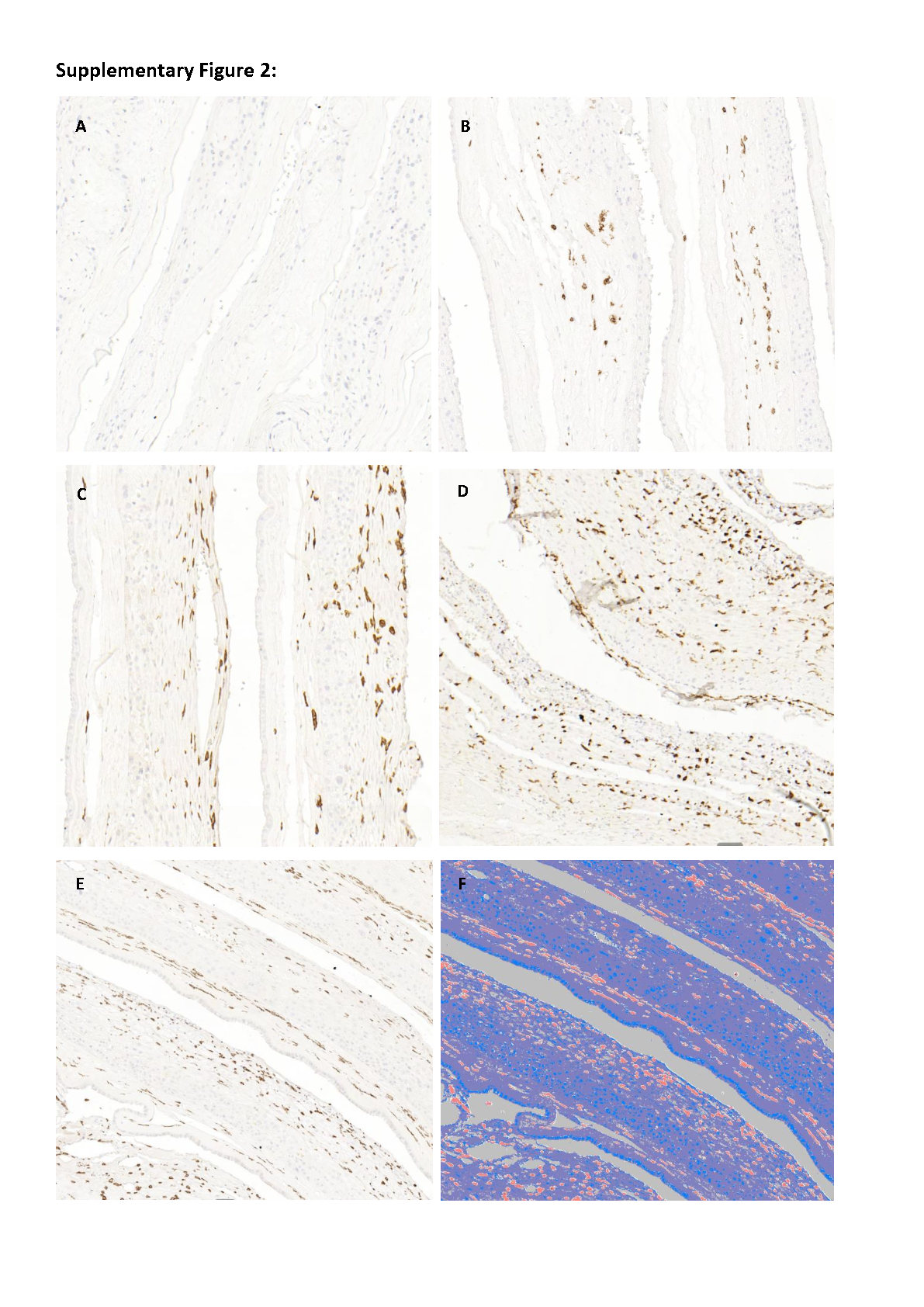

Supplement: Supplementary file 3 — Supporting Information [file AJI-87-0-s001.jpg]

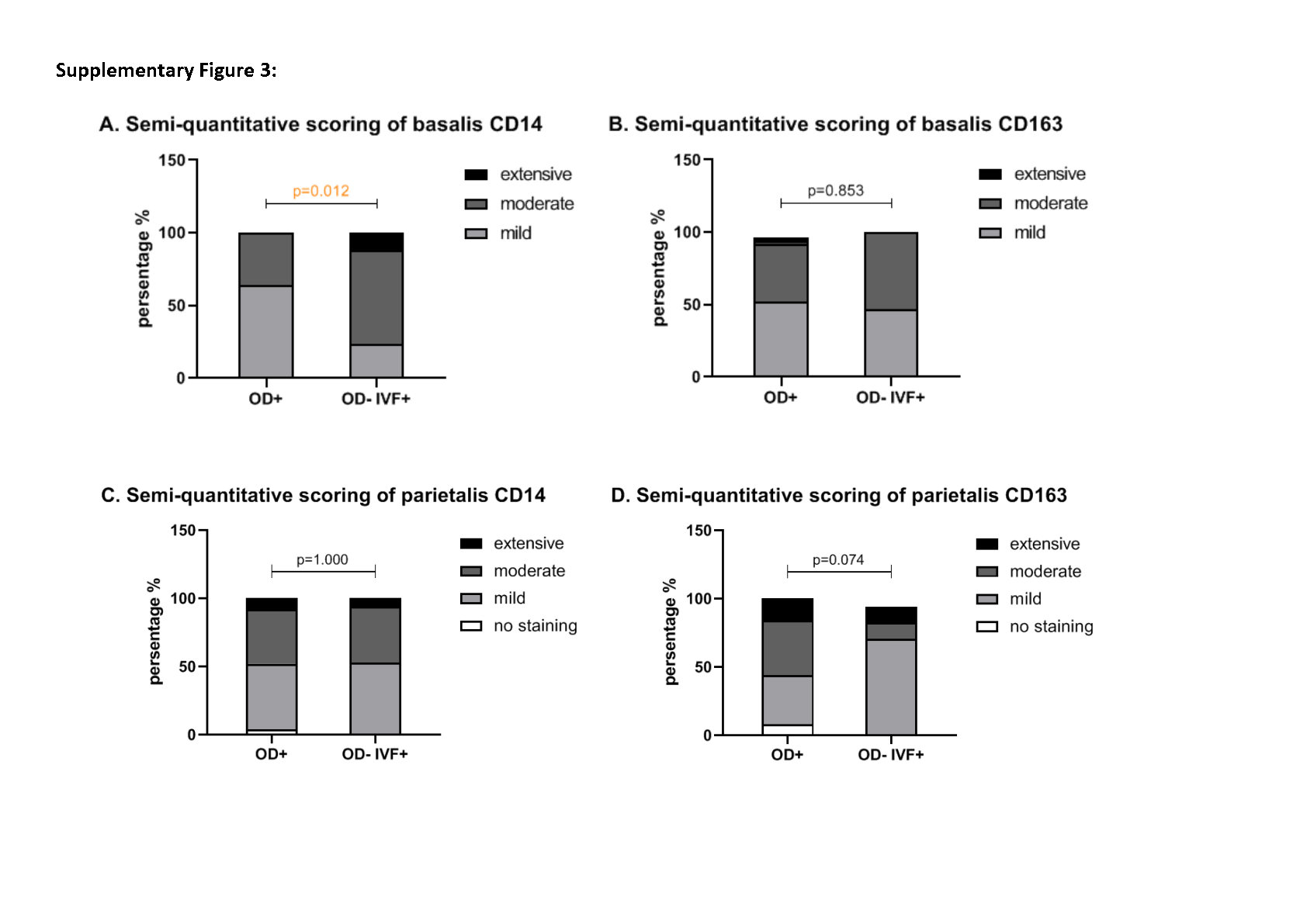

Supplement: Supplementary file 4 — Supporting Information [file AJI-87-0-s005.jpg]

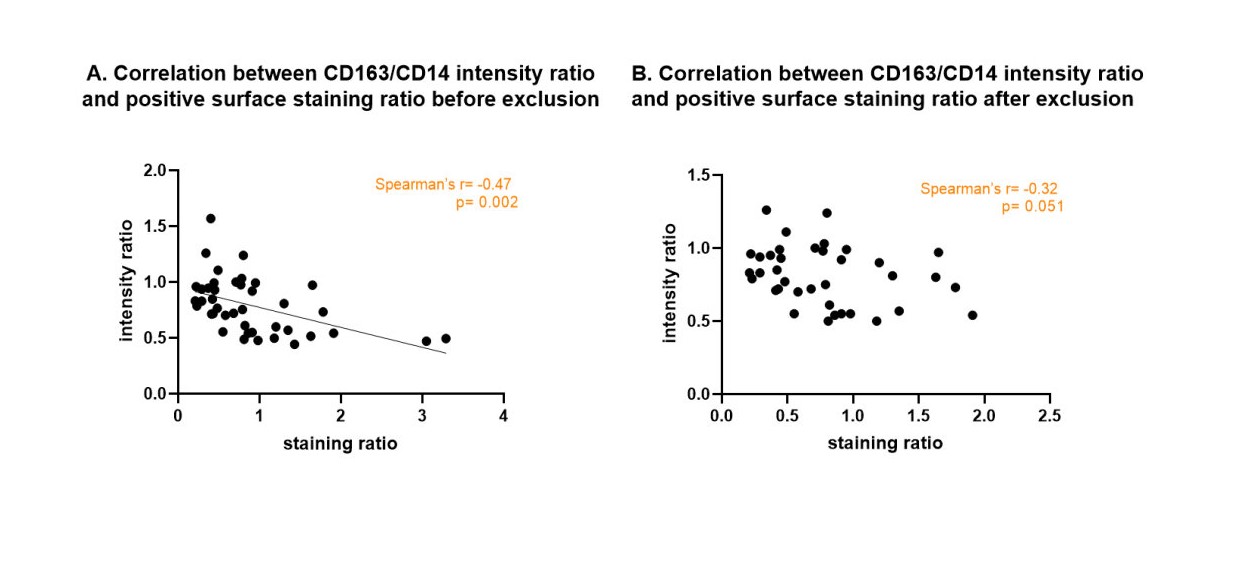

Supplement: Supplementary file 5 — Supporting Information [file AJI-87-0-s002.jpg]

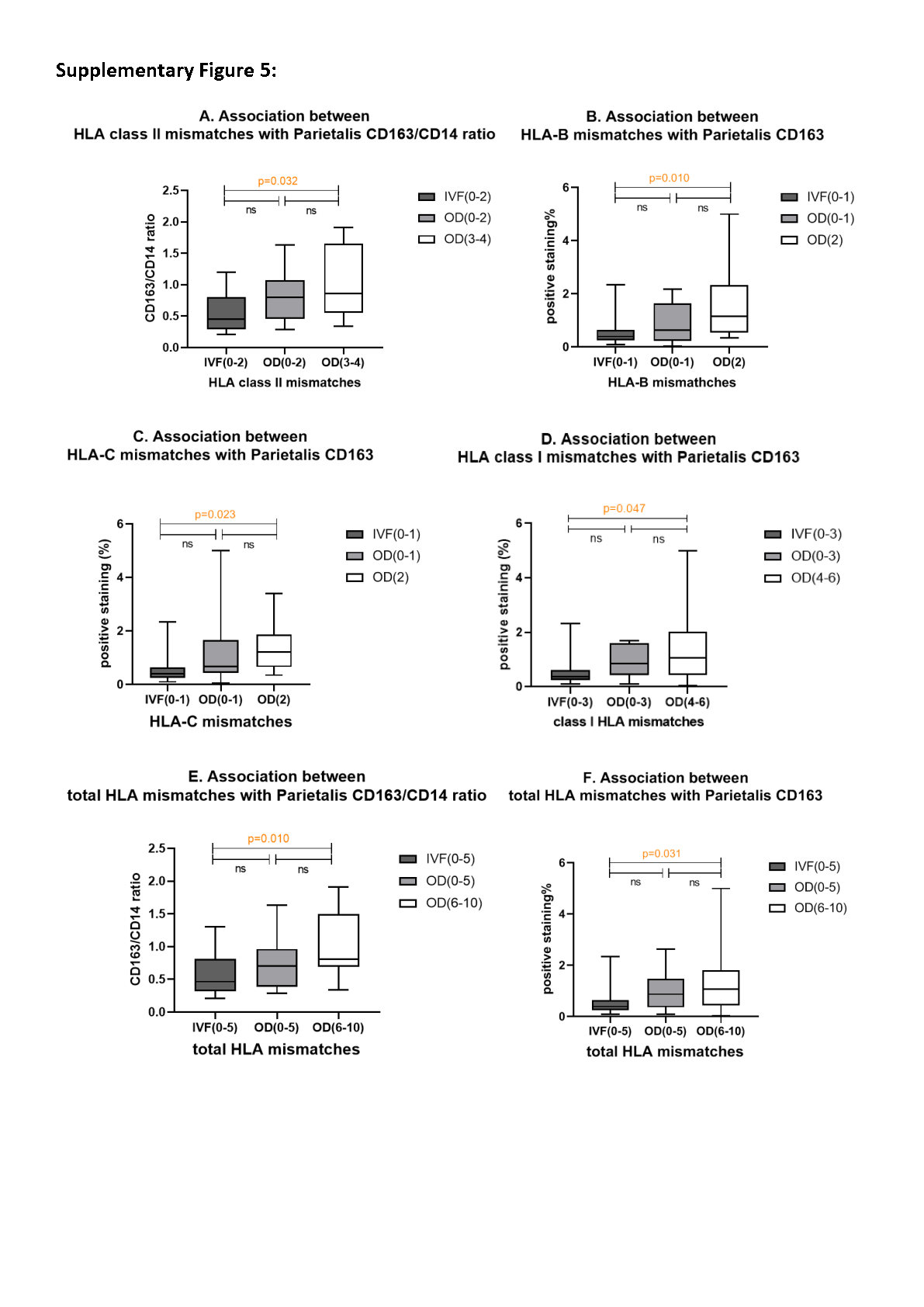

Supplement: Supplementary file 6 — Supporting Information [file AJI-87-0-s006.jpg]
